# Rethinking the ‘wellness influencer’: Medical doctors, lifestyle expertise and the question of credentials

**DOI:** 10.1177/13678779241307032

**Published:** 2025-01-06

**Authors:** Rachel O’Neill

**Affiliations:** Department of Media and Communications, 4905 London School of Economics, London, UK

**Keywords:** wellness, wellness influencers, medical influencers, plastic professionalism, dietary prescriptions, dietary cacophony, The Doctor's Kitchen, The Food Medic, Tim Spector, ZOE

## Abstract

Medical doctors are central to existing networks for wellness content on Instagram in and beyond the UK, yet remain overlooked within scholarship concerned with this terrain. Taking a case study approach, this article examines three such figures prominent in the UK context. In doing so, it challenges existing conceptualisations of the ‘wellness influencer’ and argues for an expanded understanding of the category, one that does not assume its representatives are ‘lay’ persons without recognised expertise in health and well-being. The analysis developed demonstrates that credentials cannot resolve the problems associated to wellness influencing as a genre and, as such, the entry of medical doctors to the ranks of social media celebrity may not be the ‘fix’ it is often imagined to be. Ultimately, I argue that medically qualified wellness influencers extend doctors’ role in the ‘regulation of lifestyle’, and make the case for attending to their ideological operations.

## Introduction

Within existing networks for wellness content in the UK, specifically those focussed around food and diet on Instagram, medical doctors are major players. Such individuals command large followings, sharing advice using established formats and generic conventions, as with the abidingly popular ‘What I Eat in a Day’ video. They actively collaborate with high-profile wellness influencers, appearing as guests on their podcasts and social media channels as well as trading product endorsements and speaking alongside one another at events. Several of these figures have achieved wider reach in the British media, releasing books under the wellness imprints of major publishing houses and making regular TV and radio appearances, from light-hearted features for breakfast shows to hard-hitting documentaries in primetime slots. They are aided in such endeavours by talent representatives, featuring as they do on the books of legacy media agencies as well as newer digital-first companies. Most monetise their followings, including through sponsored content and brand partnerships as well as their own commercial ventures. In short, medical doctors have not only become pivotal actors within the UK wellness arena, but have become wellness influencers *in their own right.*

While the emergence of medically qualified social media influencers has attracted journalistic commentary and discussion within the medical profession, it has been largely overlooked by media and cultural studies scholars, including those concerned with the wellness realm. My own attention was drawn to such figures while undertaking an ethnographic project on the evolution of wellness culture in the UK, focusing especially on its dietary dimensions. Closely caught up with the aspirational economies of social media, dietary wellness has become the locus of a new cultural-commercial-corporeal formation in Britain over the last decade, helmed by glamorous young influencers and entrepreneurs. Like many other arenas of influencer culture, it is a highly feminised terrain, the content of which is predominantly fashioned for and consumed by women. Though initially I did not recognise medically qualified wellness influencers as a distinct grouping within this arena, I came to do so precisely because they contravene commonplace assumptions within existing academic literature on wellness influencers. Most especially, and as I demonstrate further below, they challenge an accepted understanding of wellness influencers as typically or necessarily ‘lay’ persons who do not hold recognised credentials in the very subject – health and well-being – about which they offer advice. I thus came to wonder: why have medically qualified wellness influencers gone unexamined when they are such key actors in this arena? Does the content they produce differ from that circulated by their more conventional counterparts? And to what extent, if at all, do their credentials ameliorate the problems said to attend wellness influencing as a field that commonly inspires concern and draws criticism?

I approach these questions via a case study approach, examining content produced by three medically qualified wellness influencers prominent in the UK context – three among many I encountered and observed during my fieldwork. These are: Doctor Rupy Aujla, founder of The Doctor's Kitchen, a multi-media brand comprising several cookbooks alongside a successful podcast and app; Tim Spector, Professor of Genetic Epidemiology at King's College London and author of multiple popular science books as well as a cookbook; and Doctor Hazel Wallace, better known by her brand name The Food Medic, under which she has released three books and further administers a subscription service. This choice of case study subjects is informed by my wider ethnographic observations: these individuals are all prominent figures in the world of wellness; interact routinely with bona fide wellness stars; were mentioned repeatedly among my more than fifty interviewees; and have garnered wider media visibility, including news and magazine profiles as well as broadcast and podcast appearances.

This choice also accords with what Lilie Chouliaraki terms ‘the merit of example’, whereby case studies are selected so as to represent a ‘strategic group of *particular circumstances*’ (Chouliaraki, 2008: 9, emphasis in original). Aujla, Spector and Wallace all hold medical degrees, work or have worked in the UK's National Health Service (NHS), and are generally recognised as legitimate medical professionals and/or scientific experts. While not necessarily thought of as influencers, all possess Instagram followings of between four hundred thousand and nine hundred thousand, which in line with industry standards qualifies them as mid-tier or macro influencers (figures as of December 2024). Like other wellness influencers I have been tracking for the larger project, their followings are overwhelmingly comprised of users identified as female (circa three-quarters in each case). The content they dispense readily conforms to the genre of dietary wellness, including copious recipes as well as nutrition tips, tricks, advice and insights geared towards individual health-enhancement, wherein diet is framed as *the* vehicle to good health. Thus while necessarily bearing some of the particularities imparted by an ethnographic vantage, these case study subjects are not ‘pure particulars’, chosen at random, but instead demonstrate a ‘regularity in their features’ and ‘systematicity in their effects’ (Chouliaraki, 2008: 10).

The analysis that follows is based on multiple data sources collected over several years as part of my ethnography. As well as content drawn from Aujla, Spector and Wallace's respective Instagram accounts, @doctors_kitchen, @tim.spector and @thefoodmedic, I have examined materials made available via their websites, newsletters and podcasts. I have paid especially close attention to books as sources where their ideas are codified at greatest length. I have also examined account analytics purchased from the influencer marketing platform trendHERO as well as articles in trade magazines and news features. Finally, I have consulted editorials on the use of social media by medics in medical journals and reviewed the professional guidelines to which these sources often refer. While engaging an interdisciplinary range of scholarship germane to its topic area, this article positions itself within a body of work concerned with influencer cultures and ecologies, much of it forged by feminist scholars. It does so by reappraising what have become taken-for-granted assumptions about who and what constitutes a wellness influencer, as a specific subcategory within the ever-expanding ‘influencer industry’ (Hund, 2023).

The discussion proceeds as follows. In the next section, I consider how existing literature on wellness influencers – a still-developing area of research – conceives its object of study. I contend that a number of interrelated assumptions have perhaps too readily foreclosed the category, in ways that make medically qualified figures virtually unrecognisable as wellness influencers. Central here is the aforementioned assumption that wellness influencers are generally unaccredited and, further, that they tend to operate at a distance from or in opposition to conventional medicine, and that their popularity is attributable to collapsing public faith in the medical profession. Considering what difference credentials make in the context of wellness influencing, in the first of two empirical sections I examine constructions of expertise. Here I explain how my case study subjects flexibly deploy their medical credentials, arguing that this amounts to what I denote as ‘plastic professionalism’. In this they reproduce a central tendency for which wellness influencers are often criticised, namely an idiosyncratic approach to health advice in which personal experience prevails alongside and, at times, above licensed expertise. In the second empirical section, I examine the dietary prescriptions Aujla, Spector and Wallace make available, evidencing that their advice is both broadly similar and similarly contradictory to that dispensed by other wellness influencers, and as such is liable to add to rather than dispel confusion about precisely what it means to eat ‘well’. Concluding that medical credentials do not fundamentally alter the nature of wellness influencing or resolve the problems said to attend it, I end by discussing how attention to the figures studied here, and others like them, might help us extend current thinking on wellness influencers and the cultural-commercial-corporeal formation they have helped foment.

While based in UK examples and taking seriously the specificity of the British context, this article is nevertheless intended to raise questions of broader relevance. A pertinent reason for doing so is that the entry of doctors to the ranks of social media celebrity is not limited to the UK; indeed, the handful of papers that exist concerning the operations of what have variously been denoted as ‘celebrity physicians’ and ‘medical social influencers’ stem from contexts as diverse as China (Chen, 2018), Egypt (Atef et al., 2023) and Romania (Moraru, 2022). Influencer marketing firms further attest to this global scope, with a search for ‘medical doctor’ on sites such as StarNgage throwing up examples from across the Global North and South. It is thus readily apparent that medically qualified influencers of various kinds – within the wellness genre but also more broadly – are part of a transnational phenomenon, manifest across a range of contexts. Moreover, it is a trend that social media platforms appear intent to capitalise upon, with YouTube hosting its first ever Health and Wellness Summit in London on 18 May 2023, to which a tranche of medics were invited. It is therefore important for media and cultural studies scholars to develop an agenda for research in this area. The present paper provides one entry point to that larger project.

## Conceiving a category: Who and what is a wellness influencer?

That wellness influencers do not have recognised qualifications in the subject about which they issue advice – health and well-being – is often presented as a cut-and-dried claim in existing literature concerned with the cohort. Instead, the argument goes, their counsel tends to stem from personal experience. For Stephanie Baker and Chris Rojek, who have written extensively about wellness influencers under the broader mantle of what they term ‘lifestyle gurus’, such figures can be understood as ‘unlicensed natives’, ‘ordinary members of society, who possess limited, or no certified qualifications, and hence no professional standing for claiming expertise in … health’ (Baker and Rojek, 2020b: 10). Mariah Wellman characterises wellness influencers as online actors who ‘grow their following by sharing about their personal journey toward wellbeing’ (2023: 2). While some such individuals may have relevant educational backgrounds, in the form of ‘degrees or certifications in a health-related field’, it is their status as ‘experts of their own lives’ (Wellman, 2023: 4) that renders them persuasive to followers. For this reason, Wellman states that wellness influencers are an interesting cohort ‘to study in opposition’ to ‘traditional experts like medical practitioners and other health professionals’ (2023: 5). Taking this point further, Natalie Ann Hendry, Catherine Hartung and Rosie Welch posit that professional credentials are not only less important than personal experience for health and wellness influencers, but can actually undermine their credibility, as first-hand testimony is accorded ‘a stronger truth for legitimacy’ (Hendry et al., 2021: 437).

While the absence of recognised qualifications on the part of wellness influencers is at times indexed simply as a typical feature, in many cases this absence is figured as a *lack*, with continual references to their ‘self-appointed’ (Baker, 2022a: 4) and ‘unregulated’ (Baker and Rojek, 2020b: 7) status. Claiming that their use of the term ‘natives’ is ‘not intended to be pejorative’, but instead meant ‘to highlight the forms of authority and influence based upon experience and folk wisdom rather than formal, certified training’ (Baker and Rojek, 2020b: 5), Baker and Rojek nevertheless imply that the advice these figures dispense is in some way illegitimate. For example, when they contend that wellness influencers lack ‘any *objectively adequate certification of probity*’ (Baker and Rojek, 2020b: 12, emphasis added), this not only suggests that there are impartial and unbiased means by which expertise can be affirmed, but that offering advice in the absence of such qualifications is in some way dishonest or indecent. This logic is again in evidence when the same authors impress that few wellness influencers ‘have the certified credentials *required* to give health advice’ (2020b: 6, emphasis added). The resulting narrative is very much one of (lay) influencers versus (professional, accredited) experts, and moreover one in which the latter are accorded greater legitimacy.

A related contention is that wellness influencers operate at a distance from and even in opposition to conventional medicine and, as such, exhibit a worrying tendency to propagate health misinformation. In making such arguments, several authors alight on the case of Belle Gibson (Baker and Rojek, 2020a, 2020b; Baker, 2022b; Khamis et al., 2016; Wellman, 2023), an Australian wellness influencer who notoriously fabricated a diagnosis of incurable cancer and falsely claimed to have managed this through dietary means. While understandable considering her *cause celébrè*, the recurrent focus on Gibson nevertheless creates an impression that antagonism to allopathic authority is commonplace among wellness influencers; that wellness influencers are, at best, highly selective in how they engage with medicine, if not outright anti-medicine. Such concerns took on heightened salience amid COVID-19, where the propensity of influencers at large and wellness influencers in particular to propagate health misinformation came to the fore (Baker, 2022a, 2022b; Burt-D’Agnillo, 2022; Topham and Smith, 2023). Indeed, wellness influencers became almost mimetically linked to health-related conspiracy theories during the pandemic, an association further cemented by the popularisation of the concept of ‘conspirituality’ (Beres et al., 2023).

Bridging from these claims, an overarching argument on the part of some scholars is that the contemporary popularity of wellness influencers symptomises widespread mistrust of experts generally and medical professionals especially. For Baker and Rojek, the rise of ‘lifestyle gurus’ such as Gibson emerges from and exploits wider ‘unrest regarding organized medical practice already present in the Zeitgeist’, part of a moment where ‘disquiet about the role of professionals, especially in medicine, is at a high point’ (2020a: 397). Also referencing the Gibson case alongside other examples, Susie Khamis, Lawrence Ang and Raymond Welling argue that the influencers they study – all of whom belong to the wellness realm – exemplify ‘the mercurial dismantling of what were once “knowledge monopolies”’, such that those without formal accreditation can now ‘assume the role historically reserved for highly trained specialists (such as doctors, dieticians and scientists)’ (2016: 205). The very existence of wellness influencers is thus explained by and attributed to the prevailing force of what Baker and Rojek (2020a, 2020b) describe as a ‘low trust’ society, wherein public faith in expert institutions and groupings – especially medicine and medical professionals – has been fundamentally compromised.

Implicitly and at times explicitly suggesting that the ‘real’ experts are necessarily those who hold recognised qualifications, academic literature on wellness influencers exhibits a rather defensive stance towards certified authority. This tendency can be placed in relation to what Heidi Zimmerman aptly describes as wider anxieties surrounding ‘threats to credentialled expertise in the digital age and fears of feminised media culture’ (2021: 184). That is to say, the desire to assert clear boundaries between ‘self-styled’ and ‘actual’ experts takes shape in a context where such distinctions have become increasingly difficult to uphold. Yet there is much to be lost from defining our object of study – the wellness influencer – too narrowly, not least at such an early point in the development of scholarship in this area.

In setting up a clear opposition between wellness influencers and medical professionals, existing literature largely overlooks the existence of wellness influencers who are medically accredited. Not only this, but it makes such figures *difficult to see as wellness influencers at all.* Furthermore, one might posit that the binary frame of (lay) influencer versus (accredited, professional) expert belies a somewhat unreflexive stance towards the sexist stereotypes that overdetermine the former. As feminist scholars have long pointed out, influencers writ large are very often cast as ‘vain young women’ (Abidin, 2016). Given that wellness influencing is an especially feminised field, of which the most visible exemplars embody ‘glowing femininity’ (Nicholls and Gilchrist, 2022), this applies all the more. Indeed, it may be that medically qualified figures within the wellness field have gone overlooked, in part, because they exceed the strongly gendered contours of the category. Plainly put: for many, figures such as Tim Spector – a fifty-something white man with greying hair, who possesses a medical degree, scientific track record and prestigious university affiliation – simply does not ‘look’ like a wellness influencer (indeed, this is one reason that I have included him among my case study subjects).

It is thus necessary to reassess how the wellness influencer has been conceived to date. An important task in doing so is to question the idea that credentialled expertise can resolve the problems associated with the genre, something that currently functions as a tacit belief in the literature discussed above and express recommendation in scholarship concerned with health-related fake news on social media. Evidencing this latter point is a recent systematic review which finds many studies concluding that health professionals (as well as health organisations) should respond to the problem by cultivating a greater presence online (Melchior and Oliveira, 2022: 1511). Medical qualifications are figured as essentially remedial, capable of solving complex social phenomena in and of themselves. This line of argument finds ready parallel in journalistic commentary, with some hailing the advent of medically qualified influencers – especially in the context of and after the pandemic – as a ‘remedy to the times’ (M2.0 Communications Inc, 2022) and means to ‘fight fire with fire’ (Amclarnon, 2021). Rather than accepting what we could call the remedial thesis, the present article asks what difference credentials actually make when inserted into an existing genre, namely wellness influencing. I take this up in the following sections, first, by looking at how my case study subjects construct their expertise and, second, by examining the contents of the advice they make available.

## Leveraging personal experience through ‘plastic professionalism’

In establishing their authority to offer health-enhancing dietary and lifestyle advice, Aujla, Spector and Wallace all rely heavily on personal experience. More particularly, and as is common among wellness influencers generally, they foreground their own experiences of overcoming unexpected health challenges through individual effort. Such narratives provide the opening gambit for their books and are continually rehearsed across their social media posts as well as in podcasts, interviews, features, and so forth. For Aujla, the initial impetus to think about food and diet more seriously was the onset of atrial fibrillation (AF) episodes, which saw his heart rate soar up to 200 beats a minute. Offered a choice between medication and surgery, Aujla refused both and decided to trial various alternatives, and in doing so entered ‘a new world of wellness’ (Aujla, 2017: 12). Dietary change was at the heart of this overhaul: ‘Out went cereals and toast for breakfast, in came dark leafy greens with miso, nuts and seeds’ (Aujla, 2017: 12). The results were dramatic, and his AF episodes soon ceased entirely. This experience taught Aujla the ‘immense power of lifestyle’ and ‘incredible ability of the body to ‘self heal’ if given the correct nutrition’ (2017: 13).

For Spector, a bout of unsteadiness and double-vision while on an Alpine ski-tour served as a ‘wake-up call’ (2015: 2), as he believed these symptoms could indicate a serious underlying condition, such as multiple sclerosis or brain tumour. When medical tests failed to confirm these suspicions, Spector concluded that he had likely suffered ‘a small stroke’ (2015: 1). He was later diagnosed with a fourth cranial nerve occlusion, a relatively minor condition that typically improves on its own. Over the next few weeks, however, Spector continued to experience visual impairment and developed high blood pressure. Prescribed a course of aspirin and anti-hypertension medication, his sense of self was profoundly undermined: ‘I had gone from a sporty, fitter-than-average middle-aged man to what felt like a pill-popping, hypertensive, depressed stroke victim’ (Spector, 2015: 2). From here, Spector embarked on a ‘personal odyssey’ to ‘discover the truth about food’ (2015: 2).

Detailing ‘How it all began’ in her first book, Wallace recounts the moment her father suffered a stroke at the family dinner table, from which he later died. Thereafter, Wallace became consumed by fear that her body, like that of her father, would simply ‘give up’ (2017: 10). She began to restrict her food intake and experienced amenorrhea and hair loss before family members convinced her to seek professional help. Rejecting the diagnosis of anorexia she received, Wallace resolved to ‘make myself better without medical intervention’ (2017: 10). Though her condition improved, Wallace later experienced health problems of another kind and once again decided ‘to take matters into my own hands’ (2017: 18). Setting out to ‘completely transform’ her diet, Wallace eliminated all ‘junk and processed foods’ and embraced a regimen of ‘fresh fruits, vegetables, nuts, seeds, lean meats and fish’ (2017: 18–9). She lost weight, gained energy, experienced fewer digestive problems, and saw her skin and eyes become brighter. ‘It was then’, Wallace recalls, ‘that I started to view food as medicine’ (2017: 19).

Each of the above stories very much rehearse the typical narrative of the ‘lifestyle guru’, characterised by Baker and Rojek as ‘a journey of self-discovery from illness to recovery, triumph in the face of adversity’ (2020a: 390). In much the same way as their conventional counterparts, Aujla, Spector and Wallace describe how they determined to heal themselves through dietary and lifestyle change, without recourse to conventional medical treatment. To this extent, conversion to the belief that ‘food is medicine’ is based, at least initially, on personal experience. Such narratives allow these figures to cultivate a sense of authenticity, a central currency among influencers of all kinds. At the same time, Aujla, Spector and Wallace all invoke their medical credentials as part of their self-branding. This is clearly the case with their social media handles and brand names, as with Aujla's *The Doctor's Kitchen* and Wallace's *The Food Medic*. It also encompasses the use of professional titulars; Spector, for example, includes MD and FRCP (Fellowship of the Royal College of Physicians) in his Instagram profile, while Wallace likewise denotes her possession of a BSc, MSc and MBBCh (Bachelor of Medicine, Bachelor of Surgery). All three pepper their Instagram posts with scientific terminology and PMID (PubMed Identifier) numbers, and frequently include links to published studies to support the dietary advice they offer. As practicing doctors, Aujla and Wallace routinely highlight their clinical experience by referencing patient interactions, while Spector, an academic, invokes his research experience and publication record. Each frame their status as medical experts as equipping them with unique skills, such that they can be trusted to translate the ‘wealth of science available into easy, actionable points’ (Aujla, 2017: 62), and deliver ‘good quality, scientifically backed-up advice’ (Wallace, 2017: 6).

Perhaps surprisingly given the criticism conventional wellness influencers attract for offering health advice in the absence of formal qualifications, Aujla, Spector and Wallace are not especially critical of their unaccredited counterparts. Indeed, all three are broadly supportive of wellness messaging, even as they express certain reservations. While noting concerns about ‘the “tips” given by people who are not qualified to give such advice’, Wallace adjures that ‘there's nothing wrong with a little healthy eating inspiration’ (2017: 42). Aujla is even less equivocal. With only a glancing reference to the frustration he feels seeing ‘non-medically trained, self-styled, health “gurus”’ present advice that entails a ‘huge oversimplification of the science’, he goes on to laud wellness influencers for having done ‘an unbelievable job of motivating a generation of millennials to drink green smoothies, include kale as a staple in their shopping baskets, and exercise’, impressing: ‘Without the allure of healthy living promoted by aspirational figures, “food in medicine” wouldn’t have gained such attention in recent years’ (2017: 18). While vocally deriding what he sees as the undue authority accorded to ‘celebrities, health gurus and influencers’ (2020: 163–4) on matters of nutrition, Spector is nevertheless an enthusiastic collaborator with precisely such persons. He has appeared three times to date on the podcast of Ella Mills – widely considered the ‘face’ of wellness in Britain – and routinely features household names such as celebrity chef Jamie Oliver and TV presenter Davina McCall on his own channels.

Where Aujla, Spector and Wallace do evince a critical stance is, in fact, towards medicine itself. Even as they leverage their medical credentials to establish authority, like others in the wellness realm they very often question that accorded to the professional more generally. Such criticism takes a variety of forms. All three maintain that their education in some way prevented them from attaining the touchstone insight that ‘food is medicine’, as when Aujla relates that his GP training left him ‘horribly inept at addressing the root cause of the biggest problems facing primary care across the globe: lifestyle-related illness, including diabetes, obesity and heart disease’ (2017: 14). Ratcheting up the criticism, Spector lambasts other medics for failing to appreciate the importance of diet, claiming that too many are ‘ignorant’ and ‘out of touch’, ‘stuck in the past’ and ‘preferring checklists to individualisation’ (2020: 228–230). He cites obesity rates among medical professionals as evidence of vocational as well as moral failing, decrying doctors who would countenance leaving ‘a can of soda or a packet of crisps or biscuits on their desk’ (Spector, 2020: 229), and enjoining his audience to take umbrage at the fact that ‘We are all paying for these flawed and hypocritical services’ (Spector, 2020: 241).

In operation here is a kind of plastic professionalism, whereby formal qualifications, institutional affiliations, and so forth are invoked selectively, foregrounded in some moments while backgrounded at others. Above all, the focus is on the expertise these figures hold as *individuals*, which encompasses but is not guaranteed by their credentials. Aujla, Spector and Wallace are certainly *not* saying that doctors generally are well-placed to offer healthy lifestyle advice; indeed, Spector devotes an entire chapter of his 2020 book *Spoon-Fed* to cataloguing the profession's failings, quippingly titled ‘Don’t Trust Me, I’m a Doctor’. Rather, each contend that *they personally* are qualified to offer advice, even uniquely so. Spector in particular sets himself up as the sole arbiter of nutritional truth. Tapping into the anti-establishment sentiment conventional wellness influencers are accused of stoking, he lists doctors, government health officials, science reporters and, most notably, dietitians among those ‘unqualified’ to ‘dictate the best ways for us to eat’ (2020: 2–3).^1^ Wallace similarly positions herself as a pioneer, ‘the first of a new generation of doctors who see the importance of nutrition as the cornerstone of healthcare’ (2017: 14). Media framings rehearse this narrative, as with a *Forbes* profile of Wallace titled: ‘Meet the Doctor Who Actually Cares About What You Eat’ (Murray-Serter, 2019).

Whether or not such critiques are warranted – some doctors would argue that they are not, on the basis that lifestyle advice forms a standard component of good General Practice (Nunan et al., 2021) – the more salient point here is that they are *strategically useful* in allowing Aujla, Spector and Wallace to shore up their own authority. Thus where much journalistic commentary presents the advent of medically qualified influencers as a means to restore public faith in medicine, the manner in which Aujla, Spector and Wallace position themselves vis-à-vis the medical profession calls this into question. A possible outcome of such rhetorical manoeuvres – and an ironic one in light of arguments made about conventional wellness influencers – is that these figures may actually *undermine* trust in medicine, continually highlighting what they regard as the profession's failings while showcasing their own superior expertise. In the next section I consider how this plays out in relation to the dietary prescriptions these figures make available, demonstrating how here too they reproduce problematics associated to wellness influencers generally.

## The narcissism of small differences and its implications for ‘dietary cacophony’

The dietary ethos Aujla, Spector and Wallace each espouse aligns with that of other prominent wellness influencers, who promote a deeply classed and highly gendered orientation towards food centred on personal responsibility (O’Neill, 2021). There are repeated enjoiners to get ‘back to the basics’ and educate oneself to ‘make the right choices’ (Wallace, 2017: 20). Food is framed as the source of immense pleasure, something that should be ‘delicious and easy’ (Aujla, 2017: 70), while at the same time nutrition is a constant imperative: ‘every meal is an opportunity to nourish your body’ (Wallace, 2017: 19). Ideas of denial and restriction are expressly disavowed, as a healthy diet ‘needs to be flexible and sustainable’ (Wallace, 2017: 19). Notions of self-discovery are repeatedly invoked, with calls to ‘tune in more to our own body's needs’ (Spector, 2020: 237) and ‘find what works for you individually’ (Spector, 2020: 232). While significant inputs of time and money are required, there is an insistence that this way of eating is ‘attainable for everyone’ (Aujla, 2017: 17). Related to this are promises that, while necessitating a great deal of planning and preparation, once ‘good habits’ have been established the whole regime ‘becomes effortless’ (Aujla, 2017: 66). Indeed, cooking is framed as ‘a type of mindfulness’ (Aujla, 2017: 66), such that the labour it requires is not labour at all.

In terms of their actual contents, the dietary prescriptions set out by Aujla, Spector and Wallace are again wholly familiar, with all three endorsing a plant-based and whole food approach centred around ‘real’ and ‘natural’ foods. For Spector, a ‘good robust message’ – adapted from food writer Michael Pollan, a touchstone in the wellness domain – is to ‘*eat a diverse diet, mainly plants, without added chemicals*’ (2020: 232, emphasis in original). Wallace evinces a similar stance, advising a diet composed of ‘the most natural, unrefined and unprocessed, whole-food ingredients’ (2017: 8). Aujla issues a parallel injunction: ‘Remove refined food from your diet’ (2017: 63). All three advise maximising vegetable and fruit intake, going well beyond the standard ‘five-a-day’ guidance derived from World Health Organization guidelines. Aujla stipulates at least two different vegetables at every meal, ideally of different colours (2017: 62), while Wallace raises the bar to three, again of different colours and including at least one leafy green (2017: 46–9). A recognisable repertoire of ‘superfood’ ingredients are touted for their health-enhancing properties, even as the label itself is queried; for Aujla, these include hemp seeds, cacao, quinoa, matcha, flaxseeds, berries and seaweed (2017: 50–53), all wellness staples that also feature on Wallace's ‘shopping list’ (2017: 76).

Beyond this broad consensus – which itself largely accords with the Mediterranean diet, widely-cited for its health benefits – the dietary advice these figures prescribe is highly contradictory. Wallace and Spector, in particular, appear at odds on a range of issues. Wallace bases her nutritional advice around the concept of macronutrients, wherein food can be divided into three main groups: proteins, carbohydrates, fats. Spector is a fierce critic, contending that this represents a ‘centuries-old misunderstanding’ and going so far as to invoke the spectre of eugenics by likening it to ‘classifying all humans as African, European or Asian, and then recommending standard treatments and finding differences in health, strength or intellect according to these categories’ (2020: 6). Where Wallace accords to the view that nutritional requirements vary by sex (2017: 46) – and has made sex-based differences central to her brand identity, culminating in her most recent book *The Female Factor* (2022) – Spector dismisses such distinctions as ‘daft’ (2020: 10). Wallace accedes to NHS guidelines advising everyone in the UK to take a vitamin D supplement over the winter. Citing his own research on the topic, Spector contends not only that vitamin D supplementation ‘does not work’, but that ‘the risks outweigh any benefits’ (2020: 57). Further divisions break down between the two over the use of artificial sweeteners, with Wallace claiming that these are ‘considered safe and relatively healthy’ and may be useful for those ‘trying to lose weight’ (2017: 68), whereas Spector contends that they may ‘trick you into gaining weight’ (2020: 72). Thus Wallace lists stevia as a ‘baking essential’ (2017: 76), while Spector gravely intones: ‘Stevia may be a natural plant – but so is hemlock’ (2020: 71).

All three come into conflict over organic production. For Wallace, the choice is obvious, a ‘no-brainer’: ‘Pesticides are designed to kill organisms, so why eat them if you can choose not to?’ (2017: 248). Aujla endorses a ‘SLO’ (seasonal, local, organic) approach, acknowledging that many agricultural compounds have adverse health impacts, but contends that it is more important to get ‘the right type of food onto our plates’ (2017: 55). Advising followers to ignore ‘irrational scaremongering’, he invokes a wellness mainstay by proclaiming faith in the body's ‘powerful inbuilt “detoxing” abilities’ (Aujla, 2017: 55). By contrast, Spector warns that those who eat healthily are liable to ‘have higher levels of pesticides and weedkiller in their blood and urine than those with poorer diets’ (2020: 223), and so should try to limit their exposure by buying organic or growing their own produce. Another area of disagreement is the relative importance of size and weight as indices of health. Aujla embraces the ‘health at every size’ paradigm, contending that ‘good health, contrary to popular belief, is independent of size and especially weight’ (2017: 41) – while also promising that those who adhere to his advice ‘will lose weight’ (2017: 34). Spector, meanwhile, laments that obesity is ‘not yet classified as a disease’ (2020: 230), and warns that the ‘astronomical’ cost of this ‘global time bomb’ will have to be met by taxpayers (2015: 7). Again calling the trustworthiness of doctors generally into question, he locates blame for this situation partly within the medical profession, claiming that doctors in the UK today lack the skills required to ‘deal with obese patients’ (Spector, 2015: 228), and recalling an apparently simpler time when consultants would impress to such individuals that ‘there were no fat people in concentration camps’ (Spector, 2015: 8).

Beyond the broad embrace of a whole food and plant-based diet, then, there are significant differences in the dietary prescriptions these figures make available and numerous points of outright contradiction. On this basis, it is evident that medically qualified wellness influencers cannot provide any kind of definitive guide to healthy eating simply by virtue of the fact that they are credentialled. Indeed, their elevation on the cultural stage seems liable to exacerbate ‘dietary cacophony’ (Fischler, 1993, cited in Rousseau, 2015), whereby the sheer amount of discordant nutritional advice available makes it difficult to discern how to eat ‘well’. While the concept predates social media, wellness influencers are today among the most commonly identified culprits, charged with fomenting a virtual ‘deluge of conflicting advice’ (Baker and Rojek, 2020b: 104). However precise the dietary prescriptions they provide, and moreover precisely *because* these are so exacting, Aujla, Spector and Wallace may well perpetuate the same dynamic. To be clear, the fact that they diverge on the finer details of healthful eating is not at issue here. Given the sheer volume of research available, it is reasonable to assume that conflicting viewpoints can represent legitimate differences of opinion shaped by a shifting and inconsistent evidence base (though it must also be acknowledged that much nutritional science is tainted by commercial interests). Rather, the more salient point is that *wellness as a genre*, along with *platforms as a medium*, incentivise a kind of Freudian narcissism of small differences.

There are several dimensions to this. In the first instance, the need to cultivate a distinctive self-brand is such that influencers of all stripes must position themselves as having unique insights. In the case of medical influencers in the wellness realm, it seems fair to say that if such figures were to simply rehearse existing public health guidelines, they would probably not achieve large followings. Relatedly, because ideas of continual learning and discovery are paramount to the genre, a certain amount of revisionism is to be expected. Thus even as Spector appears to disagree with Wallace on the importance of sex-based differences, he nevertheless interviewed her on his podcast when *The Female Factor* came out. As well as allowing Spector to demonstrate his scientificity – engaging new evidence as it becomes available – such interchanges provide valuable exposure (in this case, enabling Spector to access Wallace's larger follower count). In addition, the imperative of continuous content creation and murky logics of algorithmic visibility are such that contradiction within the same niche can be useful. As such, it is not uncommon to see wellness influencers respond directly and indirectly to one another's posts to contest what has been said and offer contrasting viewpoints – effectively piggy-backing on content that has already gained traction. That medically qualified wellness influencers who make such posts typically include references to legitimate but nevertheless contradictory scientific evidence only makes the task of deciphering what to believe all the more challenging.

The churn that results from this continual output is therefore not a ‘bug’ that can be rectified through recourse to credentials. Instead, it is *a feature of wellness influencing itself.* By producing copious amounts of content – spanning not only social media but books, apps, podcasts, newsletters, webinars, live events and more – medical influencers in the wellness realm may contribute to the escalation of dietary cacophony rather than its amelioration. This is not necessarily because they are putting out questionable content, as conventional wellness influencers are accused of doing – though concerns have been raised about the propensity of lifestyle medicine, to which Aujla, Spector and Wallace are all aligned, to act a ‘Trojan horse’ for ‘non- or poorly evidenced practices’ (Nunan et al., 2021: 229). Irrespective of the veracity of the information shared – itself complicated by the fact that ‘the science’ is not singular – it is ultimately left up to individuals to sift through the mass and make decisions for themselves. Existing literature on wellness influencers not only fails to engage these points, but ultimately finds recourse to the self-same logics of personal responsibility – as when Baker and Rojek contend that it is only by understanding how lifestyle gurus operate that we can ‘be better informed as consumers’ (2020b: 13).

## Conclusion

What implications might this analysis have for how the wellness influencer is conceived and understood, and for how wellness culture more generally is studied? To begin with, it becomes possible and indeed necessary to more concertedly hold open the possibility that wellness influencers can and do come in different guises, some more conventional or commonplace than others. Most especially, and as I have sought to demonstrate through this selection of case studies, wellness influencers need not and arguably should not be thought of as ‘lay’ persons who necessarily act without licensed expertise. Some such individuals are medically qualified, with recognised credentials in health and well-being. To be sure, the ‘ordinary user’ archetype has some basis in reality. Many, even most, wellness influencers operate without relevant credentials, offering advice based largely in their own experience. Yet this is not to say that other types or models cannot be found within this domain.

By attending to the operations of individuals in the wellness arena who hold medical qualifications, and recognising them *as wellness influencers*, we can achieve a number of things. In the first instance, we build a more empirically accurate understanding of wellness as a cultural field, and a rapidly developing one at that. In my own field site of the UK, medical doctors have become increasingly prominent in the wellness arena over the last few years, a development no doubt shaped by COVID-19. Attending this is a discernible shift in the overriding orientation of much wellness content in this context, as established influencers and entrepreneurs attempt to pitch for the middle-ground: pivoting away from the ‘woo’ that characterised the early days of wellness blogging and leaning instead towards credentialed authority and scientific nomenclature. Examining how wellness culture is already and becoming still further imbricated with conventional medicine allows us to unpick the binary framework through which the two are often cast: the former a ‘fluffy’ arena in which anecdote prevails over evidence; the latter a wholly rationale terrain governed by objective truths. In doing so we can also chronicle what might be considered an emergent *entente* or quid pro quo between these domains, as more critical voices from within the medical profession are largely drowned out by those taking up the wellness mantle, aided by algorithmic forces that favour their more upbeat and lifestyle-centred content.

Examining the operations of medically qualified wellness influencers also allows us to shift attention onto less prototypical subjects of wellness culture, including those who do not conform to the generic template of a glamorous and glowing young woman – undoubtedly wellness culture's most brightly-lit exemplar, and one I myself have written about (O’Neill, 2021). For feminist media and cultural studies scholars, there is particular value in this move, allowing us to direct our gaze beyond the feminised cultural actors who already receive critical attention and focus additionally or instead on those whose social standing and personal characteristics often allow them to evade scrutiny; hence my earlier remark about Professor Tim Spector not ‘looking’ like a wellness influencer.

Adopting this lens opens up important lines of enquiry with regard questions of value, of who gains from the proliferation of wellness culture, its massification and popularisation. It is well established in the literature on influencers at large that, for every few who make a living, legions more remain aspirational labourers, not getting paid to do what they love (Duffy, 2017). Yet this is not to say that the cultural value created in these fields is not being captured; it may well be that it is, but by other kinds of economic actors, including but also apart from the platforms themselves. Here again Spector is a conspicuous example, having co-founded the personalised nutrition company, ZOE, which successfully crested the wellness wave in part by harnessing the visibility of young women in this space, with Spector himself becoming a virtual fixture of their podcasts and social media channels. The company is now a ‘unicorn in waiting’, on track to secure £100 million in investment, much of it from venture capital. Plotting developments such as this enables us to think about how the economic value of cultural spaces largely created by women is siphoned off, and in turn place the study of wellness culture more firmly within the terrain of political economy.

Finally, while a central purpose of this paper has been to argue for an expansion of our collective conception of the wellness influencer – namely by recognising the existence of medically qualified actors within this cohort – I want to conclude by highlighting what it is we can take from this literature in developing our analyses further. Undoubtedly, one of the most concerted critiques in this body of work pertains to how wellness influencers promote an individualised and individualising conception of health, framed as a personal responsibility and moral imperative. In a word, healthism (Crawford, 1980). Arguably, when this same dictum is espoused by medical doctors it takes on a rather different valence. This is because the medical profession is defined by its social contract; its representatives have a responsibility that exceeds themselves to act for the good of their patients and of society at large. It is for this reason that the use of social media by medics for the purposes of personal promotion and financial gain has become a matter of professional debate (Dugdale and Braswell, 2021; Vidovic, 2019), and that concerns about doctors’ conflicts of interest are once again on the public agenda (McCartney, 2023). Yet doctors are also liable to wield outsized *ideological authority* on matters of health and healthcare by virtue of their professional status.

Presently in the UK, the future of the NHS very much hangs in the balance. Its founding principles – most especially that of universal provision, free at the point of access – have become largely residual cultural values under neoliberalism, unmoored from the kind of political and economic commitments needed to secure them into the future (Benbow, 2017). In this context, the existence of and influence accorded to doctors espousing wellness dictum may well have pernicious consequences. Preaching the ‘gospel of individual responsibility’ (Fitzpatrick, 2001: 7) to their legions of followers online and beyond, such figures lend a veneer of professional credibility and expert authority to healthism and the privatising principles it privileges. Extending the role accorded to doctors in what Michael Fitzpatrick terms ‘the regulation of lifestyle’, they stand poised to entrench the ‘peculiar stasis of modern society’ (2001: 158) wherein a fixation with *individual health* undermines the kinds of impulses and organisational forms that do most to ensure *collective health*. These include, of course, the NHS, but also education, housing, and the environment. As such, it is crucial that our analyses continue to foreground issues of ideology and politics, power and inequality, even as we expand the conceptual confines of who and what a wellness influencer might be.

## Notes

1. Spector's inclusion of dieticians is especially noteworthy given that this grouping undertakes more comprehensive nutritional training than do those in his own fields of medicine and epidemiology. It is also a highly feminised profession, with a workforce made up almost entirely of women, at circa 93% (Health and Care Professions Council, 2021).
